# Dataset of illness classifications in Sowa Rigpa: Compilations from the Oral Instructions Treatise of the Tibetan medical classic (*Rgyud bzhi*)

**DOI:** 10.1016/j.dib.2020.105321

**Published:** 2020-02-25

**Authors:** Wüntrang Dhondrup, Dungkar Tso, Rigdzin Wangyal, Gönpo Dhondrup, Zixuan Liu, Tashi Dolma, Yi Zhang, Tawni Tidwell

**Affiliations:** a Ethnic Medicine Academic Heritage Innovation Research Center, Chengdu University of Traditional Chinese Medicine, Chengdu, 611137, China; b Center for Healthy Minds, University of Wisconsin-Madison, 625 W. Washington Ave, Madison, WI, 53703, USA; c Mongolian and Tibetan Medicine Hospital in Haixi State, Delingha, 817000, China

**Keywords:** Disease classification, Tibetan medicine, Sowa rigpa, Etiology, Four medical treatises, *Rgyud bzhi*, Visualized graphics

## Abstract

This article shares the comprehensive dataset and five visualized examples of disease categories in Tibetan medicine, or Sowa Rigpa (Tib. *Gso ba rig pa*), translated as the “knowledge field of healing.” Sowa Rigpa is a scholarly Asian traditional medical system rigorously transmitted through canonical texts and oral teachings originating in Tibet with an extensive pharmacopeia, comprehensive treatment repertoire, and nuanced etiological explications of its nosology of diseases. This medical tradition is practiced across a broad region of Asia, particularly in Tibetan regions of China, Himalayan regions of India (Ladakh, Sikkim, Himachal Pradesh), Nepal, Bhutan, Mongolia, Russia, and recently in Europe and North America. The data herein depicts disease classifications listed in the encyclopedic compendium “Oral Instructions Treatise” (*Man ngag rgyud*) of the Tibetan medical classic, the *Four Medical Treatises* (*Rgyud bzhi*), compiled in written form during the twelfth century CE. Visualized examples depict etiological relations among diseases in five of the fifteen major categories of disease: rLung Illnesses, Béken Illneses, Pediatric Conditions, Eye Conditions and Tropical Infectious Diseases. Disease names were entered into spreadsheet format and categorized by etiological hierarchical structure. Data are written in Unicode Tibetan font to retain fidelity to entries in the classical text, with parallel columns in standard Wylie transliteration. Subsets of the data are visually depicted through a graphic platform called *Interactive Tree of Life* to demonstrate etiological associations. This dataset is the first publicly available enumeration of the specific diseases, classifications and etiological relationships from the Tibetan medical classic. The data are linked to the article entitled “Tibetan Medical Informatics: An Emerging Field in Sowa Rigpa Pharmacological & Clinical Research” (Dhondrup et al., 2020).

**Table:** Specifications Table

Subject	Complementary and Alternative Medicine
Specific subject area	Tibetan medicine, Sowa Rigpa, disease classifications, etiological relationships, *Four Medical Treatises* (*Rgyud bzhi*)
Type of data	Tables; example visualized graphics
How data were acquired	Data obtained from the “Oral Instructions Treatise” (*Man ngag rgyud*) of the *Four Medical Treatises* (*Rgyud bzhi*) Tibetan medical classic, and entered into Excel spreadsheet. Data converted into unformatted text files for visualized graphic representations
Data format	Raw, analyzed and categorized, then select subsets visualized.
Parameters for data collection	Data are organized by etiologic category and written in Unicode Tibetan font, as well as Wylie Standardized Tibetan transliteration. Select English translations provided in visualized depictions.
Description of data collection	Data obtained from textual analysis of all disease categories enumerated in “Oral Instructions Treatise “of the *Four Medical Treatises* classic, and categorized by disease pathway and etiological relationship.
Data source location	Ethnic Medicine Academic Heritage Innovation Research Center, Chengdu University of Traditional Chinese Medicine, Chengdu 611,137, P.R. China
Data accessibility	With the article
Related research article	Wüntrang Dhondrup, Tawni Tidwell, Xiaobo Wang, Dungkar Tso, Gönpo Dhondrup, Qingfang Luo, Choknyi Wangmo, Tsering Kyi, Yongguo Liu, Xianli Meng, Yi Zhang Tibetan Medical Informatics: An Emerging Field in Sowa Rigpa Pharmacological & Clinical Research Journal of Ethnopharmacology 250, 2020

**Table:** 

**Value of the Data** • This data facilitates research analyzing distinctions across disease etiological relationships and related classification hierarchies in Tibetan medicine that implicate differential diagnosis; as well as analysis of theoretical explications of categories in the text compared to actual diagnosed conditions used in current clinical practice; and historical disease trends, frequencies and identifications across the Tibetan plateau and surrounding regions. • Those who will benefit from these data include researchers in the Tibetan medical field and those working with other major scholarly Asian medical traditions. • This data can be used for further insights and development of studies by analyzing etiological comparisons between Tibetan medical and biomedical disease categories; assessing trends of disease recognition and pathophysiological pathway identification in the Tibetan medical tradition; and, combined with existing pharmacological data in Sowa Rigpa, facilitate further pharmacological analysis of formulas grouped by these disease category hierarchies. • This data is also critical for research investigating the scope of Tibetan medical disease categories and etiological trajectories – what conditions might historically have been recognized compared to contemporary times; which diagnostic and therapeutic indications are explicated; and to what degree contemporary clinical practice implements this approach. • The data can facilitate intertextual comparison with the larger corpus of other Tibetan medical commentaries and classical works, as well as cross-tradition comparisons with other medical systems throughout Asia such as traditional Chinese medicine, Ayurveda, Unani, and other Greco-Arabic traditions. • This dataset is the first publicly available enumeration of the disease classifications and etiological relationships in the Tibetan medical classic, the *Four Medical Treatises* (*Rgyud bzhi*).

## 1 Data description

**Fig. 1:**
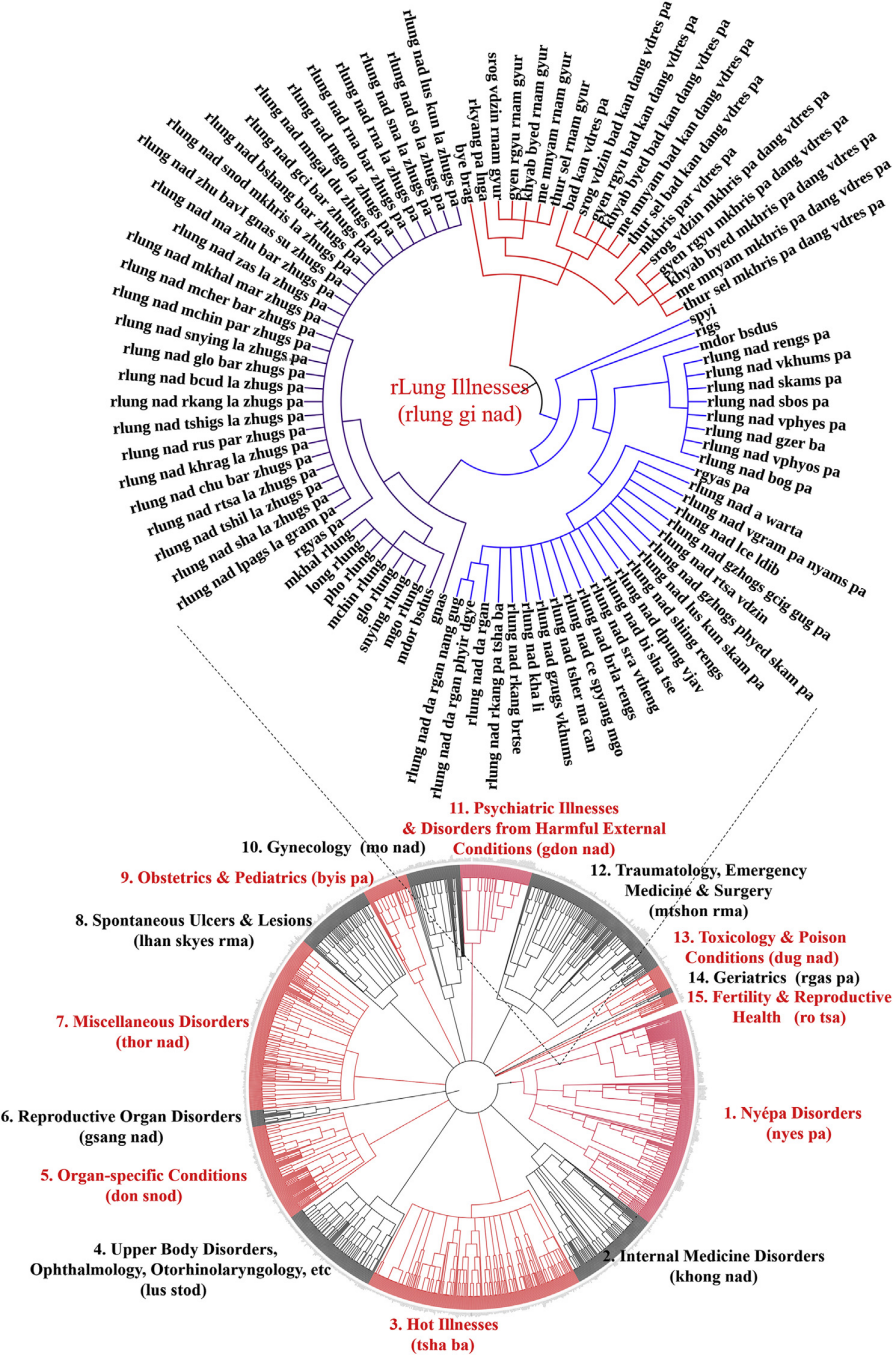
Graphic representation of all diseases enumerated in *rLung* Illnesses Category of the Tibetan medical canon as derived from “Oral Instructions Treatise” of *Four Medical Treatises.*

**Fig. 2:**
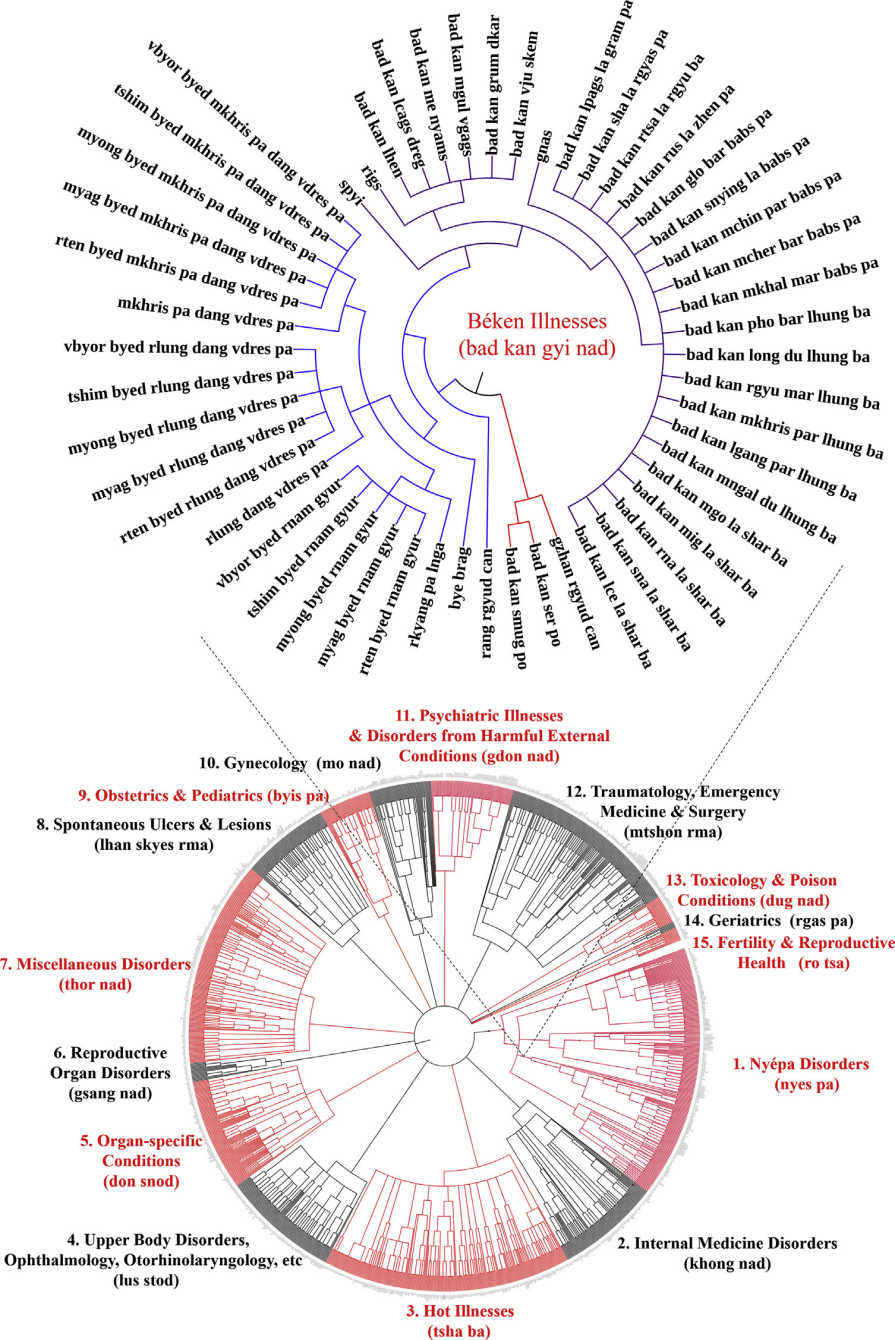
Graphic representation of all diseases enumerated in *Béken* Illnesses Category of the Tibetan medical canon as derived from “Oral Instructions Treatise” of *Four Medical Treatises.*

**Fig. 3:**
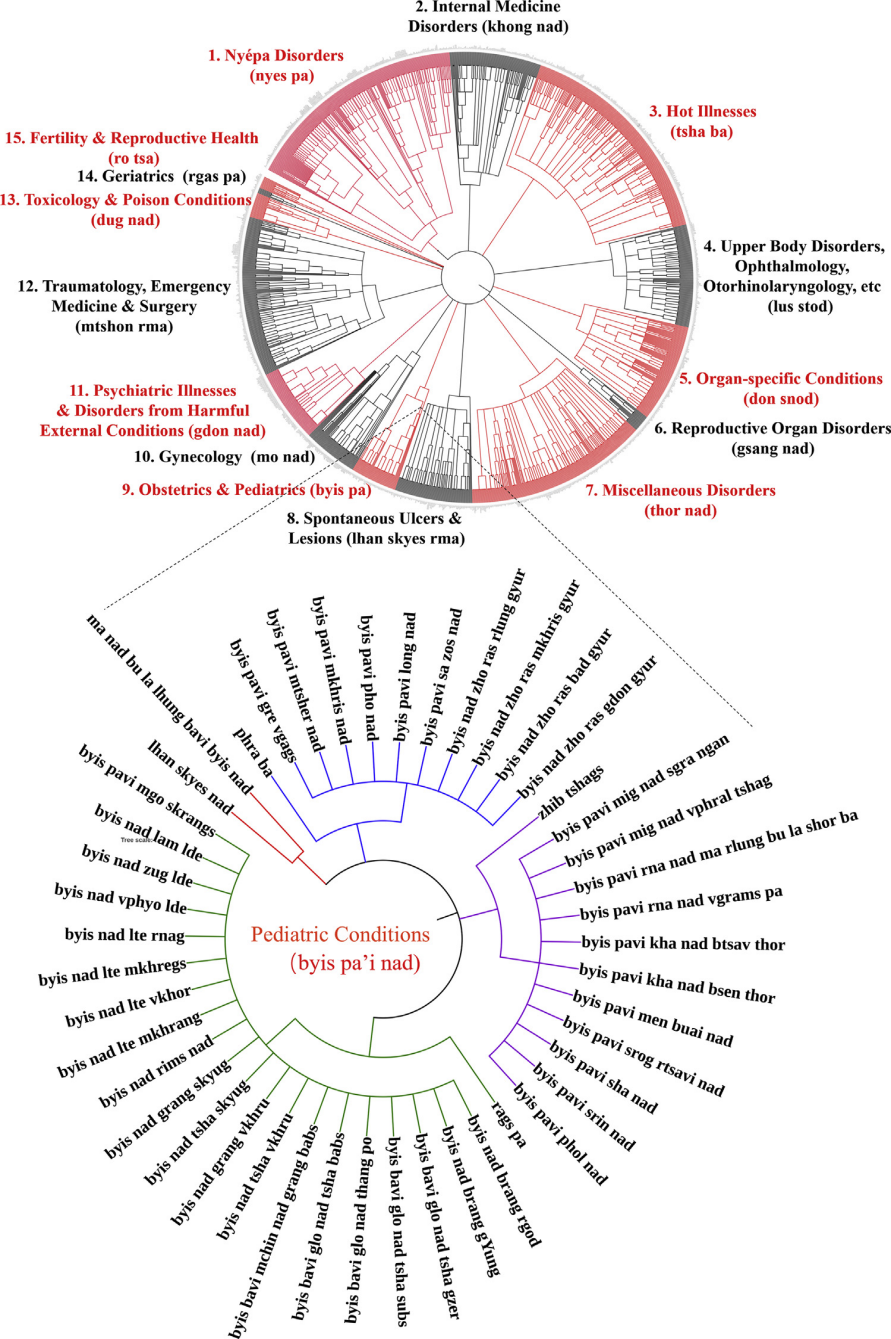
Graphic representation of all conditions enumerated in Pediatric Conditions Category of the Tibetan medical canon as derived from “Oral Instructions Treatise” of *Four Medical Treatises.*

**Fig. 4:**
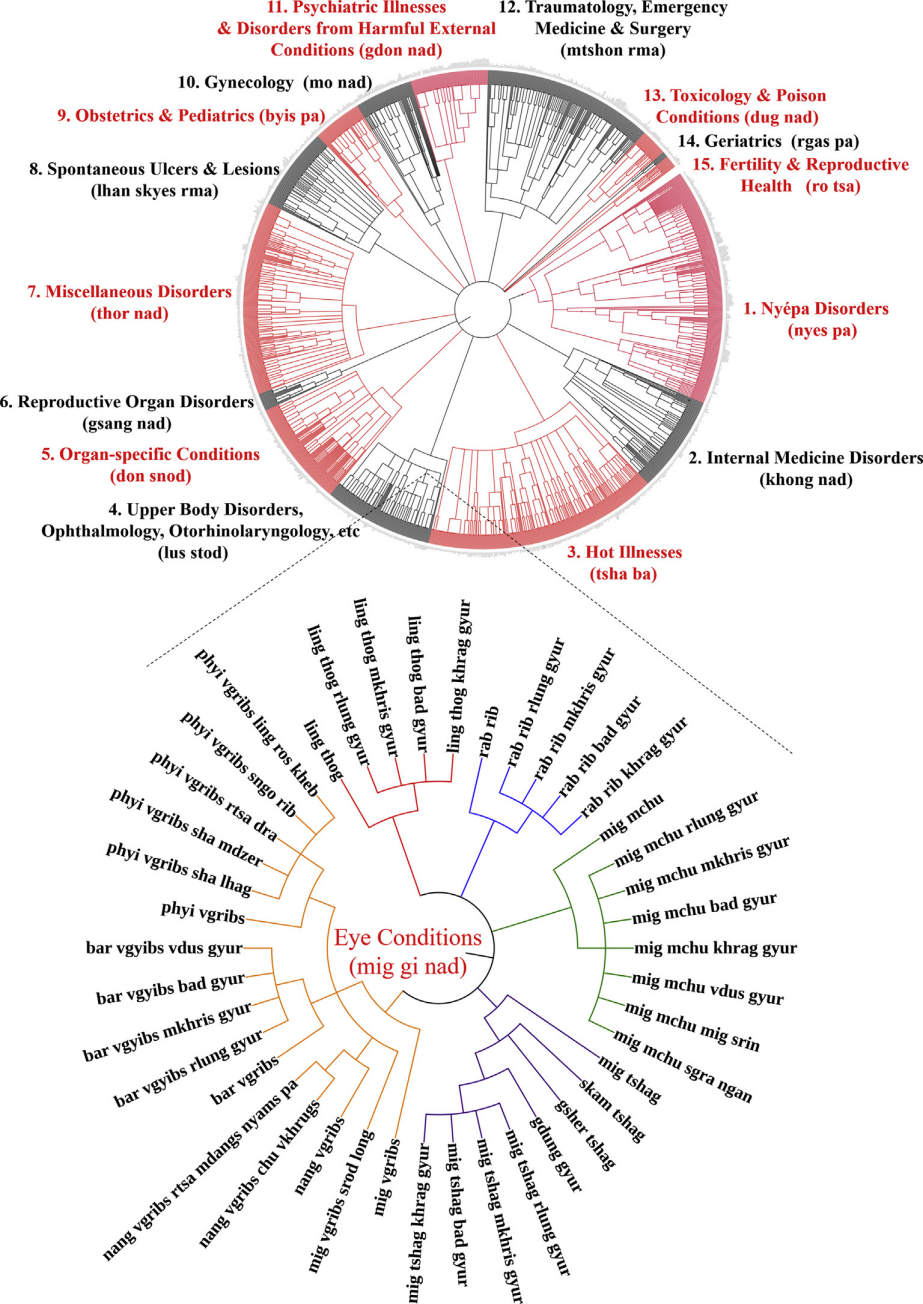
Graphic representation of all conditions enumerated in Eye Conditions Category of the Tibetan medical canon as derived from “Oral Instructions Treatise” of *Four Medical Treatises.*

**Fig. 5:**
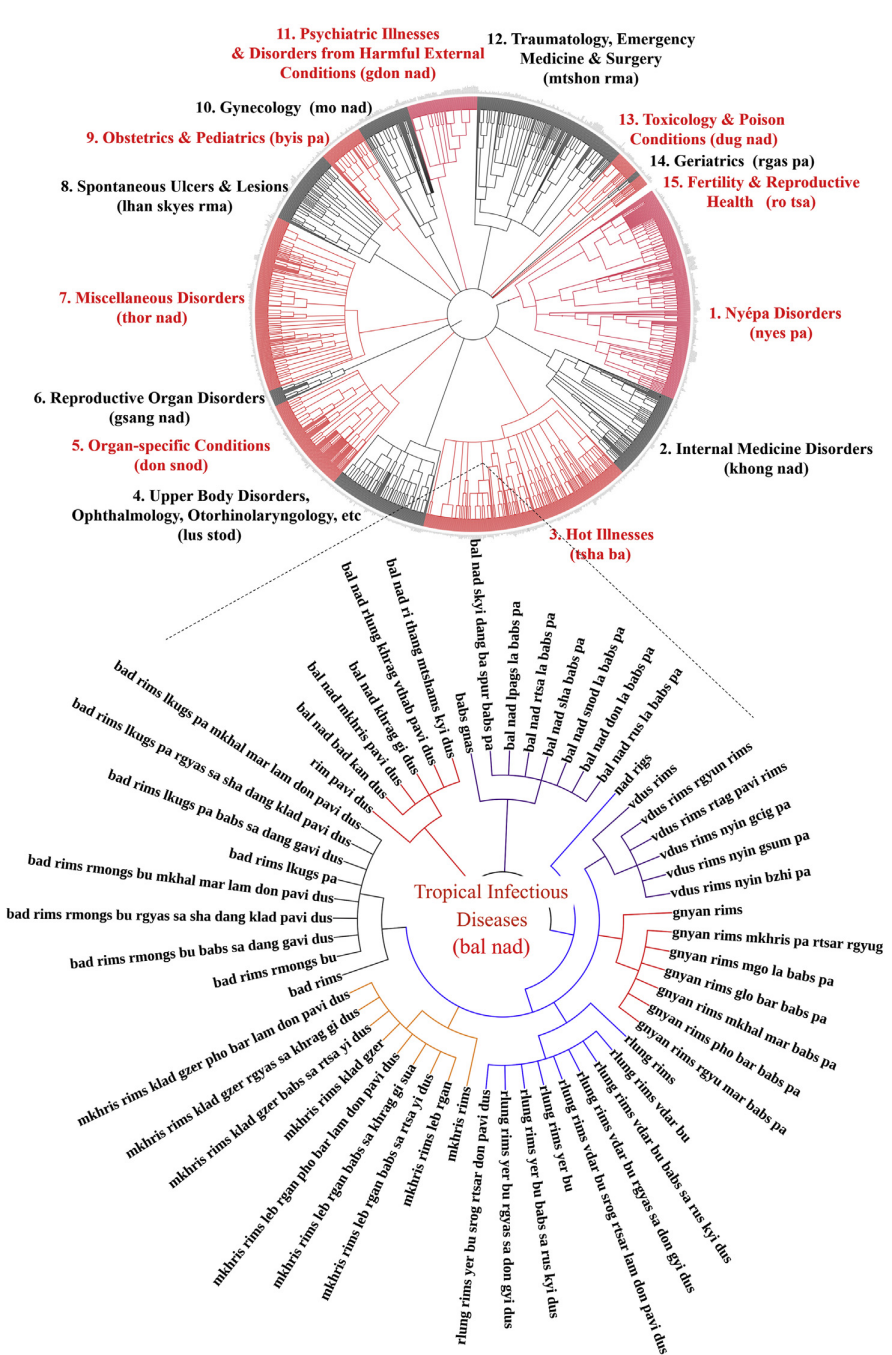
Graphic representation of all conditions enumerated in Tropical Infectious Diseases Category of the Tibetan medical canon as derived from “Oral Instructions Treatise” of *Four Medical Treatises.*

These data (Fig. 1, Fig. 2, Fig. 3, Fig. 4, Fig. 5 as illustrated applications of the attached raw data) provide a comprehensive list of the specific diseases in the Tibetan medical canon and elucidate the disease classifications articulated in the “Oral Instructions Treatise” (*Man ngag rgyud*) the finely detailed compendium of the Tibetan medical classic, the *Four Medical Treatises* (*Rgyud bzhi*). Co-authors categorized the data vis-à-vis analytics of their etiologic relationship and classification hierarchy (Fig. 1, Fig. 2, Fig. 3, Fig. 4, Fig. 5, as examples). These disease categories form the basis of differential diagnosis in Tibetan medicine, or Sowa Rigpa (*Gso ba rig pa*) translated as the “knowledge field of healing,” which is a scholarly Asian traditional medical system rigorously transmitted through canonical texts and oral teachings that originated in Tibet in the eighth century and transmitted across the Himalayas to greater central Asia. This medical tradition is practiced today in Tibetan regions of China, Himalayan regions of India (Ladakh, Sikkim, Himachal Pradesh), Nepal, Bhutan, Mongolia, Russia, and recently in Europe and North America. Compiled in the twelfth century CE, the *Four Medical Treatises* provides the foundational canon for the Sowa Rigpa tradition. Graphically representing this data has facilitated researchers in assessing distinctions across enumerations [1], between various Tibetan medical texts and within sections of the *Four Medical Treatises’* compendiums as well. Clinical diagnostic data can now map onto the theoretical categories to determine what illness types are implemented in diagnostic practice and to what frequency [[3], [4], [5], [6]]. This dataset also facilitates cross-textual analysis with other classical works in Tibetan medicine and related medical traditions, as well as illuminates historical perspectives on disease identification and prevalences. Visual representation of the data through network analysis supports assessments of theory-praxis relationships [1].

## 2 Experimental design, materials, and methods

The Tibetan medical tradition classically recognizes a total of 1616 diseases as quoted in the medical classic the *Four Medical Treatises*; yet how precisely these diseases are enumerated is still to be clearly elucidated. In order to facilitate analysis of this enumeration as well as further research on theoretical foundations and clinical applications of differential diagnostics in Tibetan medicine, researchers have compiled this dataset of the diseases and their categorical hierarchies described in the “Oral Instructions Treatise” of the *Four Medical Treatises* classic. The “Oral Instructions Treatise” section of the canon is considered the comprehensive compendium of diseases explicated in the *Four Medical Treatises*, detailing the fifteen major categories of diseases and, within each category, specifying modes of causation, diagnostic distinctions, and treatment protocols for the numerous individual diseases within each category. Researchers analyzed the “Oral Instructions Treatise” for identification specifications and modes of classification for each individual disease and developed the dataset to elucidate these identified diseases, explicate the quoted 1616 disease enumeration, and clarify the hierarchy of etiological relationships and disease classifications.

Data were extracted by textual analysis and entered into spreadsheet form as raw list (Table 1 in raw data file), and according to etiology and classification hierarchical structure (Table 2 in raw data file). Location- and type-classifications for each disease within the respective major illness category were identified as separate diseases due to distinct etiologies (see examples in Fig. 1, Fig. 2, Fig. 3, Fig. 4, Fig. 5). Thus, location (*gnas*) and type (*rigs*) enumerations are listed separately. For example, in the *rLung* (*rlung*) illness category, a class of disorders likened to neuroendocrine conditions [4], there are seven main location classifications: head *rlung*, heart *rlung*, lung *rlung*, liver *rlung*, stomach *rlung*, colon *rlung*, and kidney *rlung*. These classifications were entered into the spreadsheet separately and placed in their respective hierarchies of etiology and disease category. Such diseases can be re-analyzed according to other specific etiologic or classification relationships as well. Raw data are written in Unicode Tibetan font to retain fidelity to entries in the classical text, with parallel columns in standard Wylie transliteration [8]. Visualized graphical depictions provide English translations for the major disease categories.

To create the graphic visualizations, subsets of the data were converted to.txt files and uploaded to the data visualization platform *Interactive Tree of Life* (itol.embl.de). This platform was designed by its developers to facilitate the display, manipulation and annotation of phylogenetic trees. Because of the traditional use of allegorical trees in Tibetan medicine to display relationships across divisions, categories and classes, such as disease etiologies and nosologies, this tool provides an ideal mode to visualize the data presented herein. Use of this tool facilitates graphical depictions of the existing illnesses in a given disease category and their respective hierarchies as detailed in the *Four Medical Treatises*. Note that categories in the hierarchy, such as “location” and “type,” are drawn with separate lines in the graphical depiction as well but are not counted as separate illnesses. Only the final subcategories are enumerated as separate illnesses. Graphical methods aid analyses of illness classifications in the *Four Medical Treatises* as indicated above.

This dataset also allows for greater data manipulation power in, for example, collapsing disease category by location and looking at specific etiologic characteristics that might have similar relationships. For example, the class of diseases related to the biomedical concept of cancer all stem from etiologic relationships to interstitial fluid and poor blood quality [[2], [3], [4]]. Merging this data with clinical data, one study was able to develop an approach that creates a method for differential diagnosis of gastrointestinal endoscopic images classified into the traditional schema by looking at the endoscopic images of 300 chronic atrophic gastritis cases alongside pulse, urine, tongue and symptom records [5]. Another example using the textually-derived data alongside clinical data is a recent study [6] that characterized the drugs prescribed for classes of gastrointestinal conditions, association rules, and core standard and potential new formulae. Gongbao and colleagues [7] have used this data similarly to conduct formulation analyses for disease categories specifically related to spleen and stomach disorders.

The above figures are visualized depictions of subsets of five of the fifteen categories of disease classifications and etiological relationships from the Tibetan medical classic. The accompanying raw data provides the comprehensive list of diseases and associations.
